# Irradiation immunity interactions

**DOI:** 10.1111/1754-9485.13399

**Published:** 2022-03-08

**Authors:** David A Simon Davis, Ines I Atmosukarto, Jessica Garrett, Katharine Gosling, Farhan M Syed, Ben JC Quah

**Affiliations:** ^1^ Irradiation Immunity Interaction Laboratory, John Curtin School of Medical Research Australian National University Canberra Australian Capital Territory Australia; ^2^ Division of Genome Sciences & Cancer, John Curtin School of Medical Research Australian National University Canberra Australian Capital Territory Australia; ^3^ Radiation Oncology Department Canberra Hospital, Canberra Health Services Canberra Australian Capital Territory Australia

**Keywords:** cancer immunology, immune system, radiation immune modulation, radiation therapy, tumour microenvironment

## Abstract

The immune system can influence cancer development by both impeding and/or facilitating tumour growth and spread. A better understanding of this complex relationship is fundamental to optimise current and future cancer therapeutic strategies. Although typically regarded as a localised and immunosuppressive anti‐cancer treatment modality, radiation therapy has been associated with generating profound systemic effects beyond the intended target volume. These systemic effects are immune‐driven suggesting radiation therapy can enhance anti‐tumour immunosurveillance in some instances. In this review, we summarise how radiation therapy can positively and negatively affect local and systemic anti‐tumour immune responses, how co‐administration of immunotherapy with radiation therapy may help promote anti‐tumour immunity, and how the use of immune biomarkers may help steer radiation therapy‐immunotherapy personalisation to optimise clinical outcomes.

## Introduction

Radiation therapy (RT) is central to the management of an estimated 50% of cancer patients, either on its own or in combination with surgery and/or systemic treatment, in both curative and non‐curative settings.^1^, ^2^ Although tumour response to RT is traditionally attributed to its direct cytoreductive effect, an increasing body of evidence suggests that crosstalk between tumour and immune cells within the tumour microenvironment (TME) can play a significant role in this process.^3^, ^4^, ^5^, ^6^, ^7^ The immune system, which is also responsible for the body's defence against cancer development and progression, can either be influenced to become inactivated and ineffective or be directed to develop tumour‐promoting phenotypes by the tumour cells.^8^ Increasing evidence suggests that these tumour‐promoting immune landscapes can also limit the effectiveness of RT.^9^, ^10^, ^11^

Tumour‐driven perturbations of the immune system can affect both its innate and adaptive components, with the TME being the site and orchestrator of subsequent immunological responses.^12^ The TME is a complex milieu of cancerous and non‐cancerous cells that also include immune cells.^8^, ^13^ The interplay between cancerous cells and infiltrating immune cells significantly influences tumour development, response to therapy and consequently the clinical outcomes.^8^, ^14^ Cancer cells can manipulate the immune landscape of the TME to create a pro‐cancer microenvironment affecting treatment outcomes and prognosis.^15^ For instance, higher tumour infiltrates of regulatory T cells (Tregs) and myeloid cells are often associated with immunosuppression and correlate with tumour development and progression as well as unfavourable treatment outcomes and poorer prognosis.^16^, ^17^, ^18^, ^19^ In contrast, higher titres of some tumour‐infiltrating immune cells, such as CD4^+^ helper T cells, CD8^+^ cytotoxic T cells, natural killer (NK) cells and dendritic cells are associated with more favourable treatment outcomes and improved prognosis due to their ability to attack and destroy cancer cells.^20^

Given the increasing appreciation of the importance of the immune landscape in cancer prognosis, there is a growing need for therapeutic modalities that can rejuvenate the immune response to attack and destroy the tumour.^3^, ^21^ Exploiting RT‐mediated changes to the TME is the focus of much research, which to date has demonstrated that immune signature of the TME is a useful predictor of RT response, with tumours enriched with infiltrating T cells faring better compared with tumours infiltrated with immunosuppressive myeloid cells.^22^ Subsequently, understanding the TME immune balance and how RT affects it may be an important pre‐requisite for designing future RT courses, particularly when delivered in conjunction with immunotherapy. This review attempts to describe some of the key factors involved in the complex interplay between tumour‐driven immune effects and RT and highlights some of the potential diagnostic and therapeutic approaches to position RT to optimise anti‐tumour immunity.

## Tumour antigen‐specific immune responses and their modulation by RT

### Anti‐tumour immune responses

**Fig. 1:**
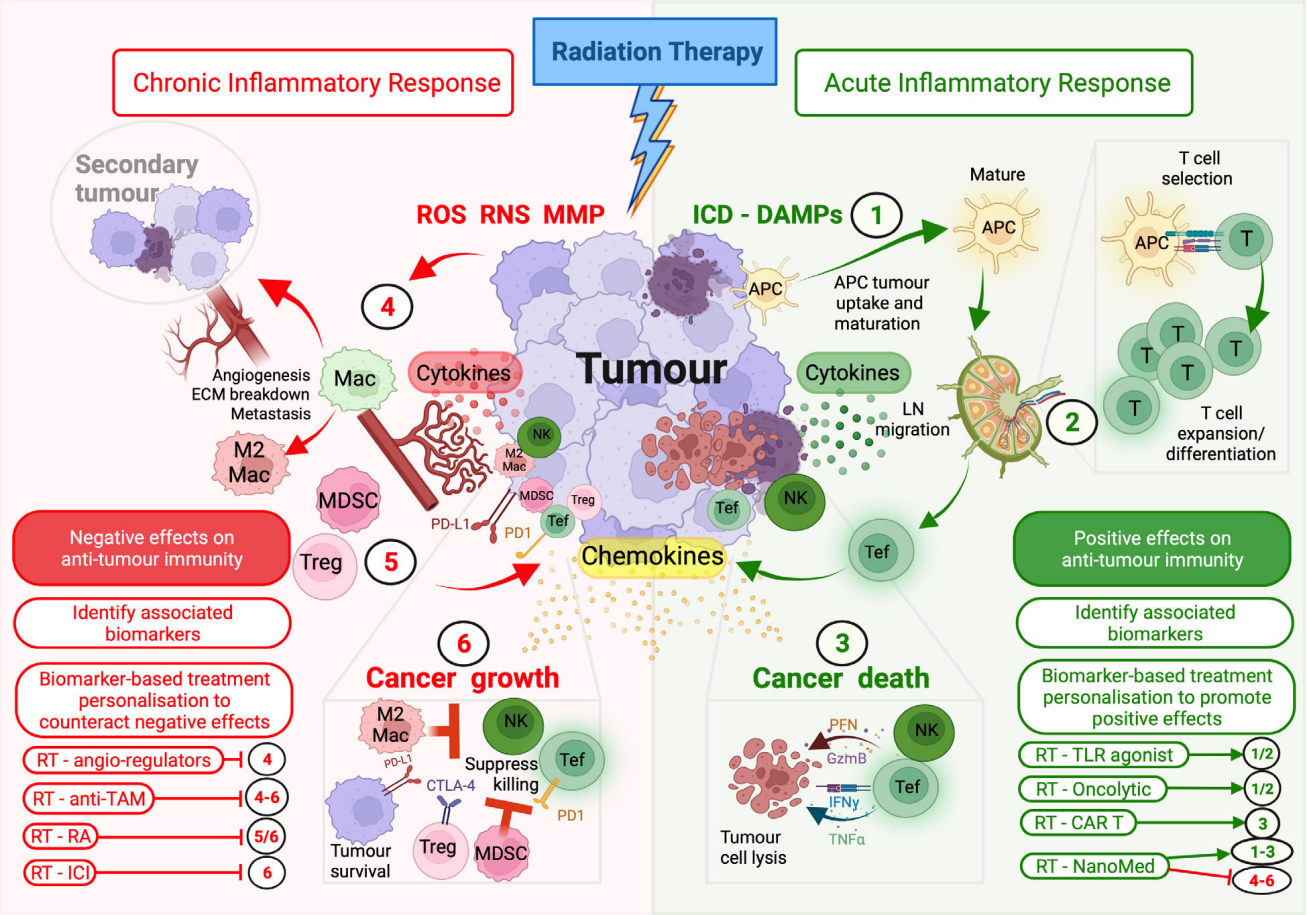
Anti‐tumour immunity and its manipulation by RT. Tumour‐specific immune responses are driven by a series of events including APC tumour antigen uptake and APC maturation (1), and APC‐mediated T cell selection and activation at the draining lymph node (2) that results in T cell expansion and differentiation into Tef (3) that lead to anti‐tumour immune responses (3). RT affects anti‐tumour immunity at the TME in several ways (green arrows indicate steps that can have a positive effect, whereas red arrows indicate steps that can have a negative effect). RT can promote anti‐tumour immunity by inducing ICD of cancer cells resulting in the release of DAMPs that aid antigen uptake and APC maturation (1), enhancing their capacity to selectively generate Tef (2 & 3). RT also improves cancer cell recognition by NK cells (3). Conversely, RT can also suppress anti‐tumour immunity and promote cancer growth and spread by enhancing tissue repair and chronic inflammatory responses via ROS, RNS and MMP effects. This results in ECM breakdown and angiogenesis that can promote metastasis (4) and the accumulation of immunosuppressive cells such as MDSCs, M2 Mac and Tregs and expression of PD‐1 and PD‐L1 (5) that inhibit anti‐tumour immune responses (6). The complex interaction of the immune system, the TME and RT, is mediated by direct cell–cell communication and via soluble factors including cytokine and chemokines and can have several consequences on tumour immunity depending on the context in which they are present. To monitor this complexity, multiple immune biomarkers may help personalise RT‐IO combinations aimed to counteract factors that have a negative effect on anti‐tumour immunity and/or promote factors that have a positive effect on anti‐tumour immunity (lower left and right panels). APC, antigen‐presenting cells; CAR T, chimaeric antigen receptor T cell; CTLA‐4, cytotoxic T lymphocyte‐associated protein 4; ECM, extracellular matrix; GzmB, granzyme B; ICD, immunogenic cell death; ICI, immune checkpoint inhibitors; IFN‐γ, interferon‐γ; LN, lymph node; Mac, macrophage; MDSC, myeloid‐derived suppressor cell; MMP, matrix metalloprotease; NanoMed, nanomedicine; NK, natural killer cell; PD‐1, programmed cell death protein 1; PD‐L1, programmed cell death ligand 1; PFN, perforin; RA, retinoic acid; RNS, reactive nitrogen species; ROS, reactive oxygen species; TAM, tumour‐associated macrophage; Tef, T effector cells; TLR, toll‐like receptor; TNF‐α, tumour necrosis factor‐α; Treg, T regulatory cells. Created with BioRender.com. [Colour figure can be viewed at wileyonlinelibrary.com]

Central to the complex immune response against cancer is the generation of tumour‐specific effector T cells, such as CD4^+^ helper T cells (TH) and CD8^+^ cytotoxic T cells (CTL), which mediate and influence tumour‐specific destruction.^23^ There are several key steps involved in the generation of effector T cells (Fig. 1), including:

1 uptake of tumour antigens by antigen‐presenting cells (APCs) at the tumour site and maturation of APCs in response to the release of damage‐associated molecular patterns (DAMPs)^24^, ^25^ that results in:
  a presentation of the tumour antigens by cell surface major histocompatibility complex (MHC) molecules,
  b upregulation of cell surface T cell costimulatory molecules, and
  c migration of mature APCs to the draining lymph node where they can interact with circulating T cells; and
2 APC selection of T cells with relevant tumour‐specific T cell antigen receptors (TCR) and subsequent activation of these T cells through costimulatory molecules resulting in proliferation and differentiation into effector T cells.

Effector T cells then migrate to the tumour site to mediate anti‐tumour immune responses (Fig. 1) where a developing or established tumour can thwart many of these steps resulting in progressive disease. RT can have both a positive and a negative effect on these processes as detailed in Table 1 and summarised below.

**Table 1:** Immunomodulatory effects of radiation therapy (RT) enhancing (shaded green) and suppressing (shaded red) anti‐tumour immunity

Component	RT effect	Function	Target cell or compartment	Consequence	Downstream immune effect	Tumour outcome	Reference
**Effects of RT enhancing anti‐tumour immunity**							
DNA damage response	Release of dsDNA micro‐nuclei from irradiated cancer cells	• Activates cGAS/STING pathway	• DCs	• Activation of NF‐κB and IRF3 transcription factors	• Type‐I interferon (IFN‐β) cytokine production	• Activation and upregulation of costimulatory molecules on DCs facilitate cross‐priming of CTLs with enhanced IFN‐γ production	5, 26, 27, 28, 29, 30
Immunogenic cell death (ICD)—release of DAMPs	Release of ATP from cancer cells	• Chemoattractant for phagocytes/APCs • Binds to purinergic receptors P2Y2/P2X7 on APCs • Activates NLRP3 inflammasome in APCs	• Macrophages and DCs	• Recruit phagocytes/APCs into the TME • Caspase‐1 dependent NLRP3 inflammasome activation in DCs	• Efficient clearance of dead and dying tumour cells prevents chronic inflammation • Mature DCs release IL‐1β and IL‐18 for efficient T cell priming	• Repopulation of the TME with phagocytes and APCs • Tumour antigen presentation by DCs for T cell priming	24, 31, 32, 33
	Translocation of calreticulin to cancer cell surface	• ‘Eat me’ signal for phagocytes • Binds CD91/LAPR on phagocytes	• Macrophages and DCs	• Endocytosis of damaged irradiated tumour cells • Uptake of tumour antigens	• Efficient clearance of dead and dying cells prevents chronic inflammation	• Source of tumour antigen for T cell priming	24, 31, 34
	Release of HMGB1 from irradiated cancer cells	• Immunostimulants for APCs • Binds TLR4 on DCs	• DCs	• Upregulates TLR4 and costimulatory molecules CD40, CD80, CD83 and CD86 on DCs • Upregulates MHC‐I expression on DCs and promotes antigen cross‐presentation	• Improved cross‐priming of T cells by DCs	• Increased population of tumour specific CD4^+^ T cells CTLs and NK cells	24, 31, 35, 36, 37
	Collective effects of DAMPs; HSP70, HMGB1 and S100A8/A9	• Activation signal for endothelial cells via TLR4	• Endothelial cells • Irradiated tumour cells	• Upregulation of adhesion molecule ICAM‐I, VCAM‐I and E‐selectin on endothelial cells • Endothelial cells release chemoattractants IL‐6, CCL7, CXCL1/KC, CXCL2/MCP‐1, CXCL8, CCL2, RANTES/CCL5, IL‐1β and G‐CSF • Irradiated tumour cell release of CXCL16	• Recruit monocytes into TME, later maturing into phagocytes and APCs • Influx of neutrophils into TME; RT polarises to anti‐tumour phenotype of TAN • VCAM‐1 and CXCL16 facilitate CTLs trafficking into TME	• Increased infiltration and maturation of APCs in TME with enhanced ability to phagocytose, process and present tumour antigen to generations of tumour‐specific T cells • Neutrophil‐derived ROS promotes tumour cell death via apoptosis	24, 38, 39, 40
DNA damage response	Upregulation of NKG2DL on irradiated cells	• Activates DNA damage response pathway via ATM and Chk1 protein kinases	• Irradiated tumour cells	• Upregulation of NKG2DL on irradiated tumour cells, ligands for NKG2D on NK	• Boosts recognition by NK cells via NKG2D‐NKG2DL interaction	• Improved NK‐mediated tumour control	41
Immune activation	Direct activation of NK cells by sub‐lethal/low‐dose RT	• Activates P38‐MAPK pathway in NK cells	• Irradiated NK cells	• Increases proliferation of irradiated NK cells • Increases NK IFN‐γ and TNF‐α productions	• Expansion of NK in TME with boosted cytolytic functions	• Further killing of tumour cells in TME	42
Immune activation	Delayed TH2‐type responses associate with eosinophilia	• TH2‐type response to counteract post‐RT acute inflammation	• Eosinophils	• Upregulates signature genes associate with eosinophil lineage selection, differentiation, activation, survival, and chemotaxis; also expands CD103^+^ DCs • Eosinophil, then DCs and subsequently CTLs infiltration into the TME	• Robust CTLs priming with enhanced ability to produce IFN‐γ, Granzyme‐A/B, and perforin	• Improved RT‐mediated tumour control	43
Cancer antigen presentation	Directly increases antigen presentation on irradiated tumour cells	• Activates mTOR pathway for antigen processing and presentation on irradiated tumour cells	• Irradiated tumour cells	• Increases intracellular peptide in irradiated tumour cells	• Increases MHC‐I‐peptide complexes on irradiated cancer cell surface	• Increases CTLs clones, promotes further killing of tumour cells	44
**Effects of RT suppressing anti‐tumour immunity**							
Inflammation	Induction of pro‐inflammatory macrophages	• Activates pro‐survival macrophages • Generates pro‐inflammatory macrophages	• Irradiated macrophages	• Activates NK‐κB and upregulates Bcl‐xL • RT skews from anti‐ to pro‐inflammatory phenotype; subsequent exposure to exogenous stimuli repolarises towards anti‐ or pro‐inflammatory • Retains noticeable MMP‐2 and MMP‐9 productions; promotes angiogenesis	• Skewing from anti‐ to pro‐inflammatory macrophages • MMP‐2 and MMP‐9 may facilitate tumour metastasis and release ECM‐bound TGF‐β • Increased angiogenesis may favour tumour rebound	• Pro‐inflammatory irradiated macrophages may promote chronic inflammation • Irradiated macrophages are pro‐invasive and pro‐angiogenic, may favour tumour metastasis	45
Tissue repair and stress response	Release of TGF‐β and HIF‐1α in the TME	• Tissue repair and revascularisation	• ECM‐bound TGF‐β • Irradiated cancer cells • Endothelial cells	• Increased TGF‐β results in: (i) polarises M1➔M2 TAMs, (ii) promotes Treg proliferation and functions, and (iii) impairs CD8^+^ T cell recruitment into TME • HIF‐1α upregulates VEGF to promote angiogenesis • TGF‐β, HIF‐1α, Type‐I interferon and CCL2 facilitate MDSCs infiltrating TME	• M2 TAMs secrete Arg‐1, NOS2, COX‐2 and more TGF‐β promote tumour growth • Tregs express TGF‐β, IL‐10 and IL‐35 and CTLA‐4 to inhibit CTLs • MDSCs express Arg‐1 and NOS2 inhibits CTLs • Increase angiogenesis may favour tumour rebound	• Net immunosuppression impairs anti‐tumour CTLs, together with increased angiogenesis promote tumour survival and metastasis	46, 47, 48, 49, 50, 51
Immune suppression	Immune suppression via PD‐L1‐PD‐1 axis	• Suppress CTL functions	• Cancer cells • T cells	• RT‐enhanced CTLs provides feedback loop to cancer cells to upregulate PD‐L1 via IFN‐γ‐JAK/STAT1 pathway • RT‐upregulation of IL‐6 in TME enhances PD‐L1 expression • RT may upregulate PD‐1 on T cells	• Increase inhibition of CTLs via PD‐1‐PD‐L1 interaction	• Dampened tumour killing allow tumour escape	52, 53, 54, 55, 56

ECM, extracellular matrix; HIF‐1α, hypoxia‐inducible factor 1alpha; HMGB1, high‐mobility group box protein 1; HSP, heat shock protein; ICAM‐1, intercellular adhesion molecule 1; IFN, interferon; IL, interleukin; IRF3, interferon regulatory factor 3; JAK, Janus kinase; M1 TAM, pro‐inflammatory macrophage; M2 TAM, anti‐inflammatory macrophage; MAPK, mitogen‐activated protein kinase; MHC‐1, major histocompatibility complex‐I; MMP, matrix metalloproteinase; mTOR, mammalian target of rapamycin; NF‐κB, nuclear factor kappa B; NK, natural killer cells; NKG2D, natural killer group 2D; NKG2DL, natural killer group 2D ligand; NLRP3, NLR family pyrin domain containing 3; NOS2, nitric oxide synthase 2; P53, tumour protein 53; PD‐1, programmed cell death protein 1; PD‐L1, programmed death ligand 1; ROS, reactive oxygen species; RT, radiation therapy; STAT1, signal transductor and activator of transcription 1; STING, stimulator of interferon genes; TAM, tumour‐associated macrophage; TAN, tumour‐associated neutrophils; TGF‐β, tumour growth factor beta; TH2, T helper type 2; TLR4, toll‐like receptor 4; TME, tumour microenvironment; VCAM‐1, vascular cell adhesion molecule 1; VEGF, vascular endothelial growth factor.

### Effects of RT that enhance anti‐tumour immunity

Radiation therapy induces cancer cell death through a number of mechanisms,^57^, ^58^ some of which may enhance anti‐tumour immune responses.^59^, ^60^ Perhaps the most widely reported example of this is immunogenic cell death (ICD).^31^, ^34^ RT‐mediated ICD exposes intracellular DAMP molecules—a type of so‐called ‘danger signal’^61^, ^62^—that collectively enhance immune responses by promoting antigen presentation by APCs (Fig. 1). At the cellular level, RT induces tumour cells to express calreticulin—an ‘eat me signal’—that binds CD91 (α2‐macroglobulin receptor) on APCs, enhancing tumour cell endocytosis for antigen presentation.^31^, ^34^ RT further enhances APC endocytic activity by interfering with the CD47‐signal regulatory protein α (SIRPα) phagocytic checkpoint pathway.^63^, ^64^, ^65^, ^66^ CD47 is a marker of self*—*‘don't eat me signal’—and its loss on aged or damaged cells leads to homeostatic phagocytosis.^67^ Critically, CD47 is overexpressed in a number of tumours,^68^ and CD47 blockade has been identified as an attractive immunotherapy target.^69^ RT‐induced CD47 loss reportedly enhances immune‐mediated tumour clearance.^63^

Tumour ICD, including by RT, also results in the release of high‐mobility group box protein 1 (HMGB1) and heat shock proteins (HSP) as well as accumulation of fragmented DNA in the cytoplasm of tumour cells.^70^, ^71^ Cytosolic DNA is sensed by the cyclic GMP‐AMP synthase‐stimulator of interferon genes (cGAS–STING) pathway, a fundamental innate immune pathway that culminates in CD8^+^ T cell activation.^72^ HMGB1 and HSP are recognised by pattern recognition receptors (PRRs) such as toll‐like receptors (TLRs),^61^, ^62^ ultimately leading to the upregulation of costimulatory molecules, such as CD40, CD80, CD83 and CD86 on APCs, and the production of type‐I interferons that can also aid in T cell activation through a variety of mechanisms.^26^, ^27^, ^28^

DNA damage responses to RT also results in interferon‐γ (IFN‐γ) and tumour necrosis factor‐α (TNF‐α) production by NK cells^41^, ^42^, ^73^ and interleukin‐6 (IL‐6), IL‐12, TNF and IFN‐γ by TH type 1 (TH1) cells—typically aiding tumour cytotoxic immune responses^21^, ^74^ that boosts motility and tumour killing capacity of cytotoxic immune cells.

Collectively, these mechanisms result in enhanced tumour cell recognition by the immune system and generation of effector T cells such as CTL and TH1 cells that can help mediate tumour destruction (Fig. 1).^62^, ^75^, ^76^, ^77^

### Effects of RT that suppress anti‐tumour immunity

While RT‐induced immune modulation within the TME can generate an anti‐tumour immune response, it can also lead to immune suppression and tumour growth and spread through complex and often overlapping mechanisms.

Radiation therapy can activate tissue repair mechanisms and chronic inflammation skewing TH cells from a type 1 phenotype (TH1) towards a TH type 2 phenotype (TH2)—typically associated with a pro‐angiogenic, pro‐inflammatory and immunosuppressive microenvironment favourable for tumour growth.^74^, ^78^ Tissue repair mechanisms and chronic inflammation also trigger macrophages to travel across the extracellular matrix (ECM) into the TME to clear dying/dead irradiated cancer cells. Here, they produce matrix metalloproteases (MMPs) that can facilitate cancer metastasis.^45^, ^79^, ^80^ RT also leads to reactive oxygen species (ROS), reactive nitrogen species (RNS) and MMP accumulation within the TME promoting the accumulation of active transforming growth factor‐β (TGF‐β) and hypoxia‐inducible factor 1α (HIF‐1α), with a range of consequences in promoting immune responses favouring tumour growth.^21^, ^74^ For example, TGF‐β and HIF‐1α can induce the production of type‐I interferons and the chemokine, CCL2, which act in synergy to recruit myeloid‐derived suppressor cells (MDSCs). MDSCs inhibit immune responses in the TME via secretion of arginase‐1 (ARG1) and nitric oxide synthase 2 (NOS2).^11^, ^46^, ^77^, ^81^ HIF‐1α can also induce angiogenesis by upregulation of vascular endothelial growth factor (VEGF) expression^82^ promoting tumour growth. TGF‐β can polarise macrophages from an M1 phenotype, which typically promotes anti‐tumour responses, to an M2 phenotype that typically helps tumour growth via secretion of ARG1, NOS2, cyclooxygenase‐2 (COX‐2) and more TGF‐β.^47^, ^83^, ^84^ TGF‐β also inhibits T cell surface expression of CXCR3, a chemokine receptor important for TME infiltration,^29^, ^85^ and also stimulates the differentiation and maintenance of Tregs^86^ that inhibit T effector cells by the production of immunosuppressive cytokines, such as TGF‐β, IL‐10 and IL‐35, and expression of the immune checkpoint molecules such as cytotoxic T lymphocyte‐associated antigen‐4 (CTLA‐4).^48^, ^49^ RT can also induce upregulation of immune checkpoint inhibitory molecules such as programmed cell death ligand 1 (PD‐L1) on tumour cells and programmed cell death protein 1 (PD‐1) on CTLs, which can directly inhibit cytotoxic immune cell effector functions.^52^, ^53^, ^87^

Collectively, RT can induce conditions favouring the recruitment of TH2 cells, MDSCs, M2 macrophages and Tregs as well as upregulation of immune checkpoint inhibitors (ICIs) in the TME that can reduce the cytotoxic immune responses against the tumour^77^, ^88^ (Fig. 1).

### RT and anti‐tumour immunity: Frenemies?

In addition to RT‐mediated immune modulatory effects having either a positive or negative influence on anti‐tumour immunity, some of the induced responses reflecting the inflammatory state of the TME^89^ can have opposing effects depending on the context in which they are produced. For example, RT triggers APC secretion of IL‐1β and IL‐18, critical components of the inflammasome pathway with reported dual roles in tumorigenesis, IL‐1β and IL‐18 can boost T cell activation but also enhance the accumulation of cells with immunosuppressive phenotypes.^31^, ^32^, ^89^, ^90^ DAMPs released by irradiated cancer cells can also activate the tumour vasculature endothelium to upregulate cell adhesion molecules—such as ICAM‐1, VCAM‐1 and E‐selectin—and the production of chemoattractants—such as IL‐6, CXCL1, CXCL2, CCL7, CXCL8, CCL2, IL‐1β and G‐CSF.^24^, ^38^, ^75^, ^91^ In fact, many of these molecules are reported to have conflicting roles in tumour development because of their pro‐inflammatory functions.^89^ Although neutrophils are well documented to stimulate tumour progression,^92^, ^93^ RT‐induced inflammation can result in the recruitment of ROS‐producing neutrophils to the TME exacerbating the oxidative stress and promoting apoptosis of cancer cells that may lead to further DAMP release.^24^, ^39^

## Manipulating local and systemic immune balance to enhance RT outcome using immune co‐therapies

An equilibrium (or lack thereof) of these overlapping immune‐mediated pathways govern the anti‐ and pro‐tumorigenesis responses. Strategies aiming to tip this precarious balance present therapeutic challenges and opportunities for enhancing anti‐tumour immune responses.

The earliest indication of the existence of important synergy between RT and the immune system is founded on Mole's 1953 report highlighting that local RT could induce systemic effects resulting in tumour reduction at distant sites,^94^ coining the term abscopal (‘off target’) effect to describe the phenomenon. Preclinical evidence has now strongly and reproducibly demonstrated that the RT abscopal effect is immune driven and that it can be amplified by co‐administration of immunotherapy.^95^, ^96^, ^97^ Immunotherapies, now an established pillar of cancer therapeutics, have drawn attention to the critical role the immune system plays in cancer development and reinforced the view that the immune system can be exploited to improve patients' outcomes.^98^, ^99^

Over the last decade and a half, ICIs have disrupted the systemic treatment paradigm for several malignancies. While they can provide durable cancer control, overall, they appear to benefit only a small proportion of patients. Of the 43.63% of US cancer patients identified to be eligible for ICIs in 2018, only 12.46% were estimated to respond to the treatment.^100^ Hence, improving the outcome of immunotherapy by incorporating RT is an attractive prospect, and strategic combinations of ICIs, RT and other immunomodulators hold great promise.

Understanding the key mechanisms that govern resistances to ICIs as well as systemic effects of RT will involve a closer study of the immune contexture in an attempt to improve clinical outcomes. At the preclinical stage, this requires RT studies to be conducted in immune‐competent subjects to ensure the intricate and dynamic nature of the interactions between cancer and immune cells is preserved. Secondly, clinically relevant surrogate markers of immune modulation need to be systematically assessed. Together, this will ensure a systematic and data‐driven approach to unravelling the immune‐mediated outcomes of RT. Here, we highlight some immunotherapy approaches that may work well with RT towards generating clinically relevant anti‐tumour immunity (Fig. 1).

### Immune checkpoint inhibitors

Immune checkpoint inhibitors act by preventing the activation of the pathways, which blocks T cells from destroying cancer cells.^98^, ^101^, ^102^ These negative immune checkpoints have been successfully targeted for blockage using monoclonal antibodies including anti‐CTLA‐4, anti‐PD‐1 and anti‐PD‐L1. CTLA‐4 blockade heightens T cell‐mediated immunity by maintaining T cell activation and restoring T cell proliferation.^101^ Combining these with RT has sparked much interest because of the obvious synergy between these modalities. For example, a recent retrospective review of patients, primarily with non‐small‐cell lung cancer (NSCLC), suggests potential improvement in local control of brain metastases following treatment with stereotactic radiation therapy combined with ICI.^103^ Another review of retrospective and prospective studies suggests patients with a low tumour burden may be the ideal candidate for combination of ICI and RT approach.^104^

The toxicity profile of the combination of RT and ICI is also an active area of research. Although a systematic review demonstrated comparable grade 3–4 toxicities compared with ICI alone in central nervous system melanoma metastases, NSCLC and prostate cancer,^105^ a phase 1 study demonstrated dose‐limiting urinary toxicity with the combination treatment of RT and pembrolizumab in bladder cancer patients.^106^ Further highlighting the need to identify patients appropriate for the combined approach, it appears patients with prior immune‐related adverse events may be at a very high risk for clinically significant and persistent radiation pneumonitis following thoracic RT.^107^

### Adoptive cell transfer therapy using CAR T cells

Cell‐lysing chimeric antigen receptors (CAR)‐T cells are cancer patient‐derived T lymphocytes that are engineered to target the corresponding ligand‐expressing cancer cells.^108^, ^109^, ^110^ Promising results for CAR T cell therapy have been reported in haematological malignancies. However, the utility of this approach in solid tumours remains unproven owing to factors such as the TME's dysregulated chemokine/cytokine signature and its enrichment in inhibitory checkpoints and immunosuppressive cells, the heterogeneity of tumour‐specific antigens, T cell exhaustion and anergy, and tumour stroma that creates a physical barrier to T cell entry.^110^

Combining RT with CAR T cells presents exciting possibilities to expand the current role of RT from a bridging or salvage therapy following relapse to more sophisticated applications such as eliminating immune suppressive cells like MDSCs, Tregs and cancer‐associated fibroblasts (CAFs) that are often enriched in the TME. These approaches warrant a better understanding of the RT parameters needed to achieve these precise biological effects.^111^

### Oncolytic virus therapy

The roots of this therapy can be traced back to William Coley in 1891, when he demonstrated tumour regression in patients with sarcoma inoculated with live and inactivated bacteria.^112^ Modern oncolytic virus therapy is based on the use of genetically engineered viruses that selectively replicate in tumour tissue inducing a local pro‐inflammatory response and resulting in augmented anti‐tumour immunity.^113^ This strategy leads to the approval of talimogene laherparepvec (T‐Vec/Imlygic),^114^ a genetically modified herpes simplex virus for intra‐tumoural injection in patients with advanced melanoma and unresectable metastatic melanoma.^115^ The phase 3 study that underpinned its approval reported superior response rate and overall survival compared with GM‐CSF at a median follow‐up of 4 years.^116^ The potential for the pro‐inflammatory effect of oncolytic therapy to augment the anti‐tumour immunomodulatory effects of RT is attractive; however, intra‐tumoural administration and substantial costs of oncolytic virus therapy remain significant obstacles.^117^

### Anti‐angiogenic therapy

There is clear interdependence between inflammation and angiogenesis: tumour‐derived immune cells release pro‐angiogenic factors that promote the growth of new blood vessels, and the new blood cells facilitate the migration of immune cells from circulation into the tumour.^118^, ^119^ A reciprocal relation also exists between RT and tumour vasculature. Generation of reactive oxygen species (ROS) in the presence of adequate tumour vascularisation enhances RT efficacy.^120^

Anti‐angiogenic strategies have been evaluated based on the premise that tumours secure vascular supply through the expression of immune system derived pro‐angiogenic growth factors, such as those of the VEGF family. VEGF ligands are expressed in most solid cancers,^121^ and specific inhibitors such as bevacizumab, sunitinib and aflibercept have shown activity in certain settings.^122^ However, inhibition of VEGF signalling has largely proven to be a disappointing strategy^123^ prompting the need to further understand how the vasculature can be effectively targeted in tumours. RT also induces changes to the vasculature, though this appears to be dose dependent and is poorly understood.^124^ Moreover, VEGF expression can be enhanced by RT.^82^

Combining RT with angio‐regulators is an attractive strategy to modulate the TME by affecting angiogenesis, but biomarker‐driven systematic studies of the effect of dose and scheduling are needed before this can be translated in the clinic.

### TLR agonists

Toll‐like receptors (TLR) are pattern recognition receptors usually expressed on macrophages and dendritic cells. They play a vital role in innate and adaptive immune responses. Immunostimulatory properties of TLR agonists can enhance anti‐tumour ICD. HMGB1 modulation, for example, has been proposed as a potential strategy to improve RT outcomes, and various HMGB1‐targeted therapeutics are currently in development.^125^, ^126^ In addition, a small number of TLR activators have already been in clinical use for several years, including the TLR7 activator R837/imiquimod,^127^, ^128^, ^129^ the TLR2/4 activator Bacillus Calmette–Guerin (BCG), and the TLR4 ligand monophosphoryl lipid A (MPLA). Notably, their use remains restricted to local (including topical) applications that may restrict their potential for combination with RT, until newer modalities become available for systemic use.

### Tumour‐associated macrophage (TAM) targeting strategies

Macrophages are myeloid cells, and their recruitment at sites of injury is associated with important tissue repair functions.^130^ Macrophages can comprise up to half of the TME, and their presence has been associated with disease progression and resistance to therapy.^131^ However, at the same time, therapeutic stimulation of the pathways involved in the recruitment, polarisation and metabolism of TAMs—using agents such as antagonists for colony stimulating factor 1 receptor (CSF1R) and chemokine receptor type 2 (CCR2), or agonists for toll‐like receptors (TLR4, TLR7/8 and TLR9), CD40 and CD47—can stimulate cytotoxic T cell activation and synergise with ICI in preclinical testing.^132^ These observations make TAM targeting strategies frontline candidates to influence the TME composition and promote the immunogenic potential of RT. Early clinical trials so far, however, report modest anti‐tumour effects by targeting CD40^133^ and downright disappointing results for CD47‐targeting agents.^134^ Combination of pembrolizumab with intra‐tumoural TLR9 agonist, on the contrary, was reported to induce immune activation at the tumour site.^135^ Overall, although this approach holds promise, better biomarkers are needed to improve clinical translation of these agents.

### Retinoic acid (RA)

Retinoic acid is a steroid hormone important in regulating mucosal immunity in the gut and promoting myeloid differentiation.^136^ All‐trans‐RA (ATRA) reportedly affects the development, differentiation, apoptosis and function of immune cells.^137^ Its potential benefit in cancer treatment is best exemplified in the treatment of acute promyelocytic leukaemia (APL) where it is used to promote differentiation of immature myeloid cells.^138^ ATRA also promotes the survival of tumour‐specific CD8^+^ T cells, increases the expression of MHC‐I on tumour cells^139^ and eliminates MDSCs and promotes their differentiation, thereby enhancing anti‐tumour immunity in patients with renal cell carcinoma.^140^ Combination of RT with ATRA has shown to induce a marked increase in TNF‐α and inducible nitric oxide synthase as well as inflammatory macrophages in local and distal nonirradiated tumours in a preclinical study of colon adenocarcinoma model.^141^ The clinical utility of ATRA has been limited to APL so far, and its use in the treatment of solid tumours warrants further exploration.^142^

### Nanomedicine

Nanoparticles are synthetic material with overall dimensions in the nanoscale (<100 nm). In modern medicine, they are utilised in various clinical applications ranging from imaging to drug or gene delivery into the tumours.^143^ Nanoparticles can enhance preferential accumulation of a drug in the tumour through active and passive targeting facilitated by the abnormal tumour vasculature coupled with ineffective lymphatic drainage.^144^ They may also be used to target immune cells in circulating blood or lymphoid tissues to modulate systemic and TME immune polarisation.^145^, ^146^ Nanomedicines thus present an attractive approach to enhance anti‐tumour immune responses by the therapeutic agents listed above, especially for those agents targeting the innate immune system, and would be an attractive RT co‐therapy.^145^, ^147^, ^148^, ^149^

## Immune signature as multiparameter biomarker to personalise RT schedules

### Rationale

Given the immune system is intertwined with cancer development^8^ and RT outcomes,^96^ immune features have immense potential as biomarkers to predict cancer progression and RT efficacy. This has implications for personalisation of future RT schedules and cancer treatments in general.^150^ While there have been several studies looking at the use of tumour biopsies to identify immune features predictive of treatment response,^151^ liquid biopsy‐based biomarkers have also received considerable attention recently^152^ as they meet many of the criteria for an optimal biomarker^153^: being minimally invasive, potentially more cost effective and easily implementable. Analysis of blood specimens would be particularly well suited for the detection of immune‐based biomarkers, since cancer growth and treatment can alter blood leukocytes and plasma immune factors.^154^ This approach would also be amenable to dynamic longitudinal biomarker sampling, to potentially allow for monitoring of disease progression and to guide therapeutic strategies throughout treatments to maximise efficacy and reduce toxicity.^155^

### Current evidence

There are an increasing number of reports on using blood immune biomarkers to predict RT efficacy in a variety of cancers, including in the treatment of glioblastoma,^156^ head and neck cancer,^157^ nasopharyngeal carcinoma,^158^ non‐small‐cell lung cancer,^159^, ^160^ oesophageal squamous cell carcinoma,^161^ hepatocellular carcinoma,^162^, ^163^ cervical squamous cell carcinoma^164^ and rectal cancer^165^, ^166^ (Table 2) Although there appear to be some common prognostic markers in these studies, such as blood myeloid cell features typically correlating with poor prognosis, it is also clear that the number of potential cancer outcome‐associated blood immune features is vast.^96^, ^167^ This area is, therefore, of increasing interest and presents research opportunities to define and optimise the use of immune parameters in the clinic.

**Table 2:** Blood immune biomarkers for RT response predictions

Cancer	Treatment	Immune biomarker	Outcome	Reference
NPC	IMRT	SII, PLR, NLR and MLR	↑ SII, NLR, PLR, MLR associated with ↓ OS	158
HNSCC	IMRT	Treg and CTLA‐4/PD‐1 expressing CD4^+^ T cells	RT‐induced changes	157
NSCLC	CRT	SII, NLR and PLR	↑ SII, NLR and PLR associated with ↓ OS	159
NSCLC	RT and antiCTLA4	Interferon‐β and blood T cells clones	Predictive of responses	160
GBM	Partial brain RT and TMZ	SIRI	↑ SIRI associated with ↓ OS	156
ESCC	dCCRT	NLR	↑ NLR post dCCRT associated with ↓ OS	161
CSCC	CRT	Lymphopenia	↑ Lymphopenia associated with ↓ OS	164
HCC	3D‐CRT or IMRT	MDSCs	↑ MDSCs associated with ↓ OS and early lung metastasis	162
HCC	CRT or SBRT	sPD‐L1	↑ sPD‐L1 associated with ↓ OS	163
Rectal cancer	SC‐RT and TME	MDSCs and Tregs	RT‐induced changes	165
Rectal cancer	CRT and surgery	VEGF, PIGF, IL‐8 and IL‐6	Treatment‐induced changes; ↑ PIGF associated with ↓ disease	166

3D‐CRT, three‐dimensional conformal radiation therapy; CRT, chemoradiotherapy; CSCC, cervical squamous cell carcinoma; dCCRT, definitive concurrent chemoradiotherapy; ESCC, oesophageal squamous cell carcinoma; GBM, glioblastoma multiforme; HCC, hepatocellular carcinoma; IL, interleukin; IMRT, intensity‐modulated radiation therapy; MDSCs, myeloid‐derived suppressor cells; MLR, monocyte lymphocyte ratio; NLR, neutrophil lymphocyte ratio; NPC, nasopharyngeal carcinoma; NSCLC, non‐small‐cell lung cancer; PlGF, placental‐derived growth factor; PLR, platelet lymphocyte ratio; SBRT, stereotactic body radiotherapy; SC‐RT, short‐course preoperative radiotherapy; SII, systemic immune‐inflammation index (peripheral lymphocyte, neutrophil and platelet); SIRI, systemic immune response index (neutrophils, monocytes and lymphocytes); sPD‐L1, soluble programmed cell death ligand 1; TME, total mesorectal excision; TMZ, temozolomide; VEGF, vascular endothelial growth factor.

### Challenges

Perhaps the most challenging aspect of using immune features as dynamic biomarkers is their known variations in concentration in human blood and the presence of various factors that may influence their signature. Ethnicity,^168^ age,^169^ RT,^170^ chemotherapy,^171^ ICIs^172^ and tumour types^154^ can all influence blood immune features. While many of these induced changes may be important in determining clinical outcomes, detailed studies need to be performed to differentiate these from the confounders.

### Multiparameter approach, preclinical models and machine learning

As the field of immune‐based biomarkers matures, there is potential for identifying a few key parameters that could be useful for RT personalisation.^96^ However, in many instances, to gain the most out of immune‐based biomarkers and indeed biomarkers in general, the interaction of several features may be required.^173^ To define the scope of these, initial research in preclinical models of cancer and RT schedules may be more practical, allowing more flexibility and control over prospective aims, and is made more relevant with new state‐of‐the‐art preclinical RT platforms now being implemented.^174^ While technical advances allow several features to be extracted from biopsies for this type of research,^175^, ^176^ interpreting such multivariate data suits a machine learning approach to build models on data as it comes to hand to supplement clinical decisions.^177^, ^178^ Indeed, machine learning approaches are seen as being integral to future cancer treatment management^179^ and so a greater understanding of these approaches will be helpful as the clinical environment evolves to include these approaches of immune biomarker‐based treatment personalisation (Fig. 1).

## Conclusions

For well over a century now, RT has established itself as a highly effective cancer treatment modality in both curative and non‐curative clinical settings. Although it was recognised quite early on that some interplay exists between the immune system and anti‐tumour RT responses,^94^, ^180^ variable success of ICIs in a variety of malignancies over the last decade and a half has made the appreciation of this interaction mainstream.^181^ With a better understanding of the cancer dissemination process,^182^, ^183^, ^184^ along with expansion of more effective systemic treatments in parallel with the development of highly conformal and accurate RT delivery techniques, the indications for RT are constantly evolving particularly in the setting of metastatic malignancies.^185^, ^186^, ^187^, ^188^, ^189^ It is especially in this clinical context, a better recognition of immunomodulatory effects of RT has the potential of changing clinical practice.

Radiation therapy can have both favourable and unfavourable effects on anti‐tumour immunity, and the interactions between RT and the immune system are complex, often overlapping, and on many occasions paradoxical.^21^, ^74^ The challenges, and hence the opportunities, lie in identifying how best to manipulate and utilise these immunomodulatory effects of RT to improve treatment outcomes.

Exploitation of the potential synergism between RT and other immune‐modulating modalities in reversing the immunosuppressive effects of certain cancers on the TME and beyond warrants understanding of how best to combine these treatments, how to sequence them and how to select the optimal RT dose‐fractionation schedules and target volumes. Biologically relevant biomarkers diagnostic of tumour‐induced immune polarisation and predictive of treatment response are needed to guide the systematic evaluation of the plethora of possible combinatorial strategies leading to improved personalisation of treatments.

The survival benefits attributed to local treatments in the recent clinical studies in the setting of metastatic disease are very promising.^185^, ^186^, ^187^, ^188^, ^189^ Future studies will shine more light on whether the clinical effects are direct results of enhanced anti‐tumour immunomodulation and/or indirect outcomes of tumour cytoreduction reinvigorating the dampened immune system.

## Funding

This work is partly supported by the Radiation Oncology Private Practice Fund, Canberra Health Services; and Research and Innovation Fund, Centre for Health and Medical Research, ACT Health, ACT Government.

## Data availability

All data analysed for this study are present in the submitted files and/or stored in an institutional public repository server.
